# Sudden Onset Occipital Headache as a Presenting Feature of Ischaemic Stroke

**DOI:** 10.7759/cureus.116918

**Published:** 2026-09-25

**Authors:** Haseeb Mudassir, Safyaan Tanzeel, Yewande Odu, Wasim Ullah Khattak, Ayibongwe Sithole, Morsal Rahimy, Nawaid Ahmad

**Affiliations:** 1 Respiratory and Acute Medicine, Shrewsbury and Telford NHS Trust, Telford, GBR; 2 Cardiology, Shrewsbury and Telford Hospital NHS Trust, Telford, GBR; 3 Geriatrics, Shrewsbury and Telford Hospital NHS Trust, Telford, GBR; 4 Nephrology, Shrewsbury and Telford Hospital NHS Trust, Telford, GBR; 5 Internal Medicine, Shrewsbury and Telford Hospital NHS Trust, Telford, GBR; 6 Respiratory and Acute Medicine, Shrewsbury and Telford Hospital NHS Trust, Telford, GBR

**Keywords:** beta2 microglobulin, ischemic stroke, occipital headache, stroke, stroke-associated headache

## Abstract

Headache is an uncommon presenting feature of ischaemic stroke, and when it occurs, it is usually moderate and sustained rather than sudden and self-limiting. We describe the case of a 56-year-old man who presented after a single, abrupt, extremely intense occipital headache that reached 10/10 and resolved completely within about 30 seconds. The neurological examination on arrival was normal, including a National Institutes of Health Stroke Scale (NIHSS) score of 0. The patient separately reported a subtle change in vision on the left side, and formal perimetry showed a left superior homonymous quadrantanopia that had not been apparent on bedside confrontation testing. Computed tomography (CT) of the head was unremarkable with no acute changes, and magnetic resonance imaging (MRI) confirmed a 3.5 cm acute infarct in the right occipital lobe, with incidental small chronic infarcts in the left cerebellum and right caudate head suggesting previous clinically silent events. There were no conventional vascular risk factors apart from an untreated dyslipidaemia, and the patient had sustained a minor head injury a week earlier. An extended thrombophilia screen revealed an elevated anti-β2 glycoprotein I antibody, with confirmatory testing pending. This case is a reminder that a brief, isolated, thunderclap-type occipital headache with a normal examination can be the only warning of an occipital infarct, that the diagnosis can hinge on a patient-reported visual symptom and formal visual field testing, and that early MRI is needed because CT is insensitive to early posterior infarction.

## Introduction

Headache occurs in a proportion of patients having an ischaemic stroke, and it is reported more often when the posterior circulation is involved. In the current classification, headache attributed to ischaemic stroke is usually of moderate intensity, has no specific characteristics, and is very rarely the presenting or the prominent feature [1]. A very short-lived, severe occipital pain occurring on its own is therefore atypical, and it can steer the clinician towards benign explanations first.

This case is instructive because several things pulled against the diagnosis at once. The headache was brief and had resolved before any assessment. The neurological examination and the National Institutes of Health Stroke Scale (NIHSS) [2] were normal. Apart from an untreated dyslipidaemia, there were no conventional vascular risk factors. There had been a recent minor head injury that could easily have been blamed for the whole picture. We describe how the diagnosis was reached and set it against the small existing literature on headache as a presenting feature of ischemic stroke.

## Case presentation

A 56-year-old man developed an abrupt, extremely intense pain at the back of the head, rated 10/10, which settled within about 30 seconds and did not recur. There were no associated features of visual aura, nausea, photophobia, weakness, or speech disturbance. A short while later, the patient noticed a subtle change in vision on the left side but could not map out exactly where it was located.

A week before the event, the patient had been struck on the right side of the head with a metal bar and had remained well afterwards, with no loss of consciousness and no headache. The patient did not attend hospital after the incident.

Due to the nature of his symptoms, he was referred to Same Day Emergency Care (SDEC). On arrival at SDEC, the observations were blood pressure of 158/90 mmHg, heart rate of 59 beats per minute and regular, SpO₂ 97% on air, and temperature 36.6°C. Examination showed no focal neurological deficit. Cranial nerves, limb power, sensation, coordination, and gait were all normal, and confrontation visual field testing did not reveal an obvious defect at the bedside. The NIHSS was 0 [2], scored by confrontation in accordance with the scale's standard administration instructions; the field defect described below was demonstrable only on formal perimetry. A 12-lead ECG showed sinus rhythm.

The working differential diagnosis for a sudden severe occipital headache included subarachnoid hemorrhage, vertebral artery dissection (particularly given the recent head trauma), post-traumatic headache, and occipital (posterior circulation) infarction. The feature that pulled away from a purely benign cause was the persistent left-sided visual symptom set against an otherwise normal examination, which prompted formal visual field testing and urgent imaging.

Due to the visual complaint, an urgent ophthalmology review and formal perimetry were requested. Formal visual field assessment revealed a left superior homonymous quadrantanopia. 

Twenty-four-hour ambulatory ECG monitoring showed sinus rhythm with normal intervals and no evidence of atrial fibrillation. Transthoracic echocardiography demonstrated normal left ventricular function with a borderline dilated left atrium. CT of the head was unremarkable, with no acute intracranial abnormality. A lumbar puncture was subsequently performed to exclude subarachnoid haemorrhage after the thunderclap-type onset, given the recent head injury and the differential of traumatic subarachnoid haemorrhage; the opening pressure was normal, and there was no evidence of blood or xanthochromia. MRI of the head, performed after the non-diagnostic CT, established the diagnosis, demonstrating a 3.5 cm acute infarct in the right occipital lobe (Figures 1, 2), with no haemorrhage and normal intracranial vessels on the angiographic sequences. The scan also showed small defects in the left cerebellum and in the head of the right caudate nucleus, with appearances in keeping with old infarcts, pointing to previous ischaemic events that had been clinically silent. Laboratory investigation results are summarised in Table 1. 

**Figure 1:**
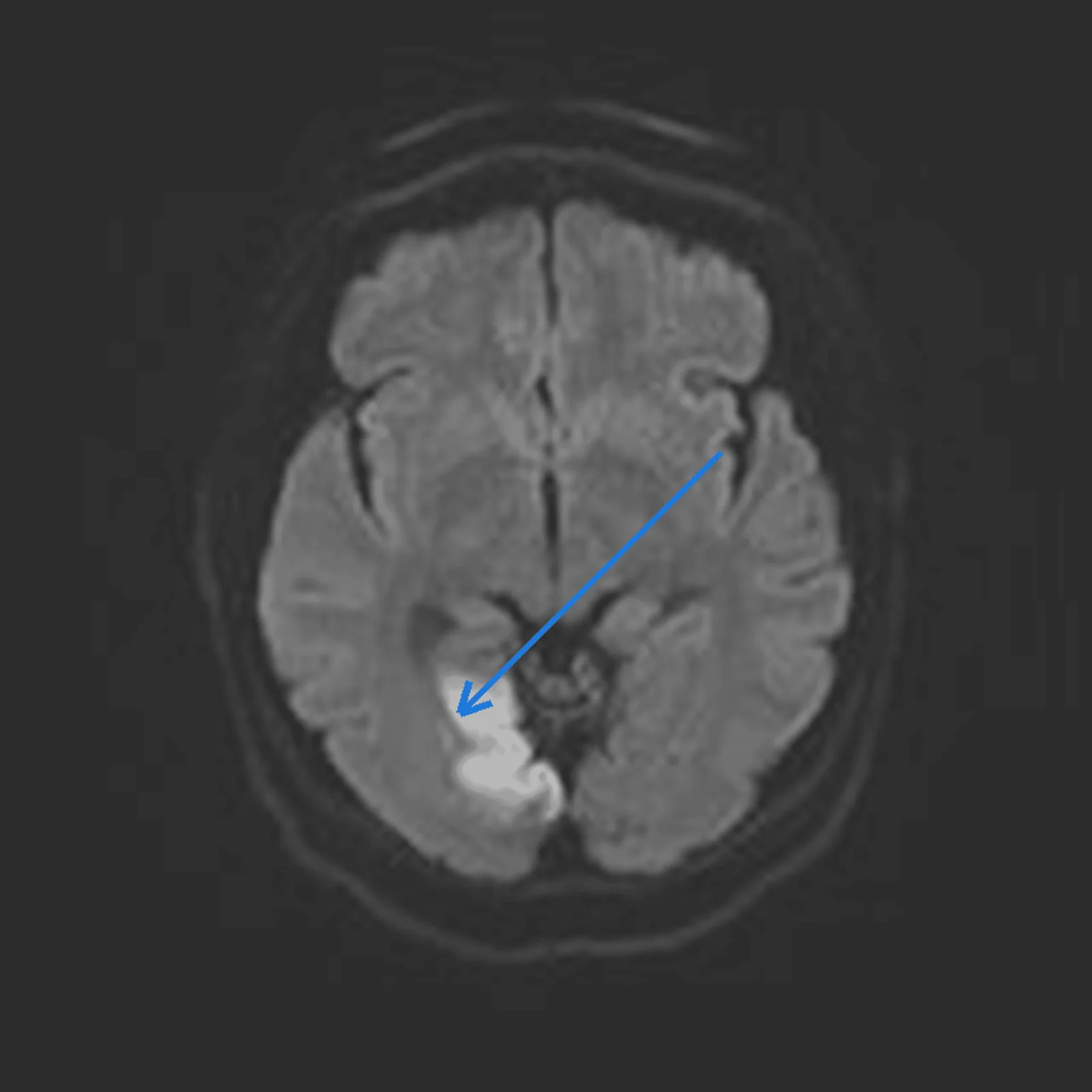
Axial diffusion-weighted imaging (DWI) MRI of the brain. The arrow indicates the 3.5 cm acute infarct in the right occipital lobe.

**Figure 2:**
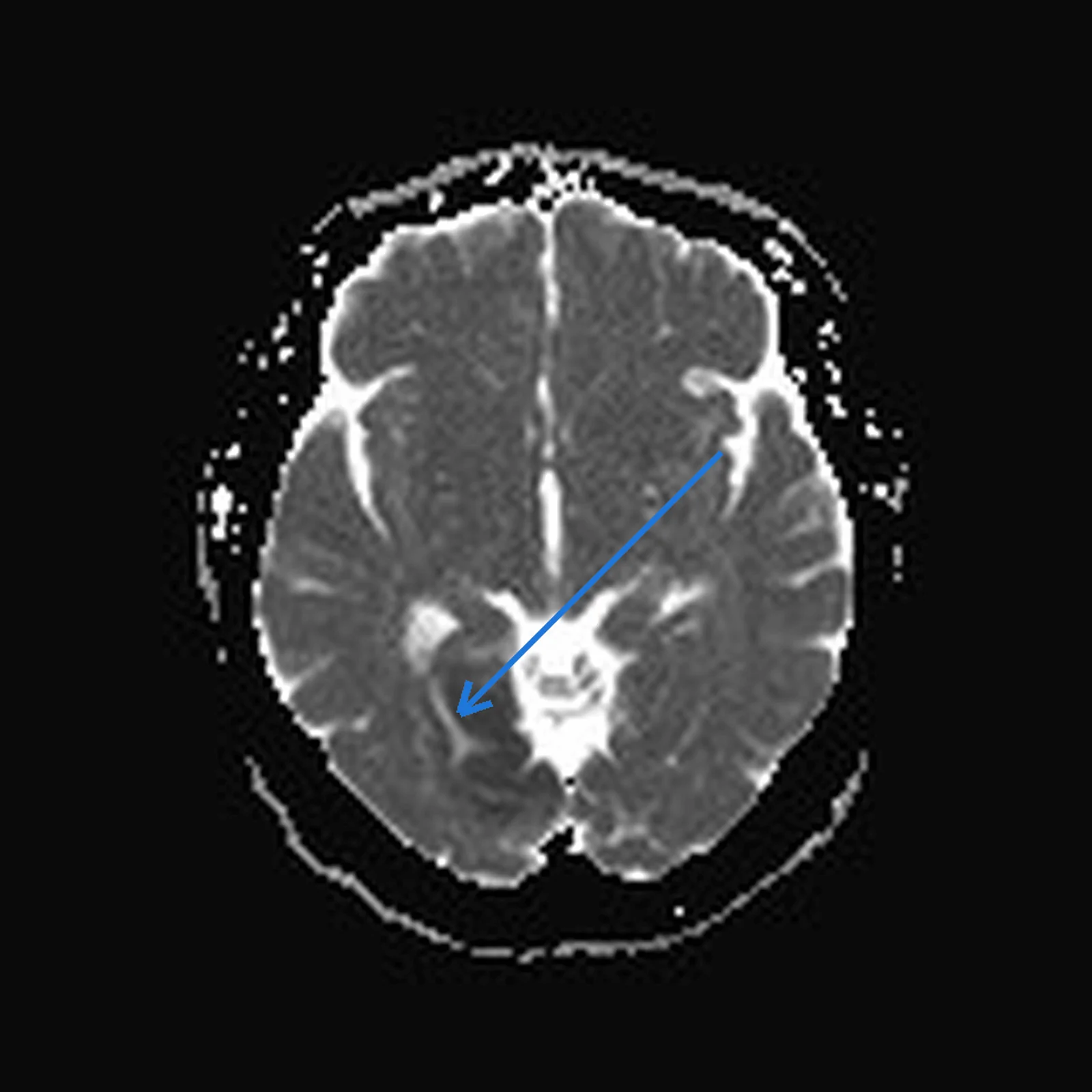
Axial apparent diffusion coefficient (ADC) MRI of the brain. The arrow indicates the 3.5 cm acute infarct in the right occipital lobe.

**Table 1:** Laboratory results

Investigation	Result	Reference range
Glycated haemoglobin (HbA1c)	38 mmol/mol	<42 mmol/mol (<6.0%)
Total cholesterol	5.9 mmol/L	<5.0 mmol/L
Low-density lipoprotein (LDL) cholesterol	3.6 mmol/L	<3.0 mmol/L
High-density lipoprotein (HDL) cholesterol	1.31 mmol/L	>1.0 mmol/L
Antinuclear antibody (ANA)	Mildly positive	Negative
Anti-PM/Scl-75 antibody	Positive	Negative
Anticardiolipin IgG	4.37 RU/mL	<20 RU/mL
Anticardiolipin IgM	<2 RU/mL	<20 RU/mL
Anti-β2 glycoprotein I antibody	39 EU/mL	<20 EU/mL

The patient had no history of hypertension, diabetes, smoking or atrial fibrillation but did have an untreated dyslipidaemia. As the Sydney classification criteria for antiphospholipid syndrome require antibody positivity to be confirmed on two occasions at least 12 weeks apart, repeat anti-β2 glycoprotein I testing was arranged to establish persistence before the result could be considered diagnostic [3].

Management followed standard secondary prevention for ischaemic stroke: aspirin 300 mg once daily for two weeks, followed by a switch to clopidogrel 75 mg once daily as long-term antiplatelet therapy, together with high-intensity statin therapy and gastric protection with a proton pump inhibitor. The patient was advised not to drive for four weeks in line with Driver and Vehicle Licensing Agency (DVLA) guidance, and follow-up was arranged in the stroke clinic alongside the outstanding antiphospholipid and cardiac investigations.

At follow-up, the headache had not recurred, and the quadrantanopia had resolved, with no further neurological symptoms. Secondary prevention was in place throughout, and the outcome of the repeat anti-β2 glycoprotein I antibody test was still awaited to confirm or exclude antiphospholipid syndrome.

## Discussion

This case illustrates how a posterior circulation stroke can present in an atypical way. Stroke-associated headache is usually described as moderate, dull, and lasting longer than a few seconds, and it is very rarely the presenting or the prominent feature of an ischaemic stroke [1]. Here, the patient had the opposite: an explosive, very brief occipital headache that had already resolved before any clinical assessment could be done.

A short-lived, severe headache that resolves and is then followed by an established infarct fits the concept of a sentinel headache, a warning symptom that precedes the stroke rather than accompanying it. In a large prospective series, headache was present at stroke onset in about 15% of patients, and a similar proportion reported a sentinel headache in the week before the stroke, yet only around 60% of sudden-onset headaches met the existing diagnostic criteria [4]. Thunderclap headache as the first and main feature of an ischaemic stroke, with a normal examination, has been reported before in embolic cerebellar infarction [5] and in a supratentorial infarct where the examination, the CT and the CSF were all normal [6]. In this case it was the visual field defect identified on formal testing, rather than the headache pattern alone, that prompted urgent MRI. We would emphasise that a brief isolated headache does not itself warrant MRI, and it would be neither practical nor defensible to image every patient presenting in this way. What altered the assessment here was a patient-reported change in vision, volunteered rather than elicited and not demonstrable at the bedside. Formal visual field assessment should therefore be considered whenever a thunderclap headache is accompanied by any reported visual disturbance, even where confrontation testing and the wider neurological examination are normal. This sequence also has practical advantages. Formal perimetry is inexpensive and widely available, whereas urgent MRI is not, and imaging on the basis of a brief headache alone would be neither justifiable nor achievable in most settings. Demonstrating a field defect first provides the indication for MRI and makes the pathway transferable to centres where access to imaging is constrained.

The recent head injury deserves its own comment. A blow to the head a week before a posterior circulation infarct raises the possibility of a traumatic vertebral artery dissection seeding emboli, and it also offers a tempting benign label of post-traumatic headache that could have anchored the assessment in the wrong place. In this case, the intracranial vessels were reported as normal, but dissection of the extracranial vessels can be missed on standard sequences, so dedicated vessel imaging of the neck should be considered where the mechanism is otherwise unexplained [7].

The two older, clinically silent infarcts in separate territories are the other thread worth pulling. Silent infarction is common and carries an increased risk of future symptomatic stroke [8], and finding lesions in more than one vascular territory makes a proximal embolic source more likely [9], which is why the workup here centred on the heart and the rhythm rather than the large vessels alone. We would emphasise that these aetiological investigations were prompted by the pattern of infarction demonstrated on MRI, not by the headache; the headache warranted imaging, and the imaging findings warranted the subsequent workup. 

The thrombophilia results add a further candidate mechanism. An elevated anti-β2 glycoprotein I antibody raises the possibility of antiphospholipid syndrome, but a single positive result is not diagnostic: the classification criteria require the abnormality to persist on repeat testing at least 12 weeks apart, and transient antiphospholipid antibody positivity is well recognised around acute events. If the repeat test confirms persistence, this would have implications for the choice of antithrombotic therapy in secondary prevention [7]. The mildly positive ANA and anti-PM/Scl-75 antibody are of uncertain relevance here, as PM/Scl antibodies are associated with scleroderma and myositis overlap syndromes rather than with ischaemic stroke.

The bedside examination is also worth dwelling on. Confrontation visual field testing is not sensitive for a quadrantic defect, and the abnormality here was only found on formal perimetry [10]. It follows that an NIHSS of 0 does not exclude a visual field deficit, since the visual field item is itself scored by confrontation. Without the patient volunteering the visual change, and without formal fields being arranged, the acute infarct could easily have been missed. The pivotal step in this case was therefore the patient's own account of a subtle visual disturbance, taken seriously in the context of a recent head injury that offered a ready alternative explanation. The localisation is consistent: a left superior homonymous quadrantanopia points to the contralateral inferior visual pathway, that is the occipital cortex below the calcarine fissure, matching the right occipital infarct described [11]. Early MRI with diffusion-weighted imaging is the key investigation, particularly because CT is insensitive to early infarction in the posterior fossa and occipital region [12,13].

The sequence of investigations is also instructive. A headache lasting seconds is atypical for aneurysmal subarachnoid haemorrhage, which characteristically produces severe and persistent pain, and once a homonymous field defect had been identified, posterior circulation infarction was the leading diagnosis. Where a thunderclap headache is accompanied by a visual field deficit and an early CT is normal, MRI should take priority over CSF examination.

## Conclusions

A patient-reported visual disturbance accompanying a sudden, severe occipital headache can be the only indication of a posterior circulation stroke, even when the headache is very brief, and the neurological examination and NIHSS are normal. We do not suggest that a brief isolated headache warrants MRI; rather, where such a headache is accompanied by any reported visual change, that symptom should direct the assessment. A short-lived headache that resolves may represent a sentinel headache, and a thunderclap onset associated with visual symptoms warrants brain MRI as the priority investigation, and MRI should not be delayed by further investigation for subarachnoid haemorrhage where the headache was momentary and the early CT was normal. Subtle visual field defects are often not obvious on bedside examination and are picked up only on formal perimetry, so in selected patients a formal visual field or ophthalmology review is worth arranging. MRI with diffusion-weighted imaging remains critical where CT is non-diagnostic. 
